# The international health elective: a stepping stone for tomorrow’s global surgeons and anaesthetists

**DOI:** 10.1007/s40037-018-0439-4

**Published:** 2018-07-13

**Authors:** Bilal Abou El Ela Bourquin, Sujit Gnanakumar, Michael F. Bath, Tom Bashford, David K Menon, Peter J Hutchinson

**Affiliations:** 1 School of Clinical Medicine, University of Cambridge, Cambridge, UK; 2 Division of Neurosurgery, Cambridge Biomedical Campus, Addenbrooke’s Hospital, NIHR Global Health Research Group for Neurotrauma, University of Cambridge, Cambridge, UK; 3 Division of Anaesthesia, Cambridge Biomedical Campus, Addenbrooke’s Hospital, NIHR Global Health Research Group for Neurotrauma, University of Cambridge, Cambridge, UK

## Introduction

Global surgery is often labelled ‘the neglected stepchild of global health’ and has been defined by the *Lancet Commission on Global Surgery *as ‘an area of study, research, practice, and advocacy that seeks to improve health outcomes and achieve health equity for all people who need surgical and anaesthesia care, with a special emphasis on underserved populations and populations in crisis’ [1, 2]. Over five billion citizens are unable to access safe, affordable, surgical and anaesthesia care with the majority of these living in low and middle income countries (LMICs) [2]. To address this, we must invest not only in training and infrastructure but also in research capacity, in order to build an appropriate evidence base for global health improvement. Partnerships between surgeons and anaesthetists in high-income countries (HICs) and LMICs have proven effective in boosting research output [3]. However, sustaining progress requires the nurturing of today’s medical students to become tomorrow’s global surgeons and anaesthetists.

Beyond surgery, the call in recent years for a greater focus on global health has been met with interest and enthusiasm by medical students across all specialities, with organizations such as Students for Global Health and GlobalSurg leading the charge for hands-on student participation in international health projects [4, 5]. However, it is within the medical curriculum that the most transformative educational experience in global health can be found, namely the international health elective. The perceived benefits of undertaking international health electives in LMICs have been well described, ranging from honing clinical examination and practical skills to greater exposure to pathologies and appreciation for the wider determinants of health [6, 7]. Considering this scope for personal and professional gain, it is no surprise that 40% of United Kingdom (UK) medical students choose to do their international health elective in LMICs [8]. The natural question therefore is whether this enthusiasm and potential can be harnessed for furthering academic global health initiatives. Academic partnerships offer a potential medium for student participation in research. We advocate that student international health electives be embedded within robust academic LMIC-HIC partnership programs to support both global surgery and wider global health efforts.

## The benefit of academic partnerships for student electives

As for all academic research, global health research projects undertaken as part of international health electives would be scrutinized by ethical committees, minimizing the potential unintended harms to the host community and the student involved. This is in contrast to the all-to-common caricature of fearlessly confident, overly enthusiastic, and culturally-insensitive student tourists disrupting the system and frequently being a risk to patients and themselves [9]. Even those with insight and the best of intentions may find themselves practising beyond their scope, propagating the idea of poor treatment for the poor and contravening the principle of ‘*Primum non nocere*’. An academic partnership between LMIC and HIC institutions offers a means by which the ethical guidelines espoused by the Working Group on Ethics Guidelines for Global Health Training, and General Medical Council requirement that students ‘work within their limits of competence’ can be adhered to [10]. We propose that bilateral student involvement in these research projects culminating in shared authorship can help alleviate the problem of international health electives being primarily a privilege of HIC students. LMIC counterparts are often not granted the opportunity to partake in electives in HICs due to administrative and financial hurdles and, in terms of research, LMIC collaborators are often unacknowledged in their authorship of publications [11]. By embedding international health electives into equitable and long-term academic partnerships LMIC students can also be empowered, and even funded, to lead research from their local institutions—helping in a small way to address some of the causes behind ‘brain-drain’ where LMIC students seek to migrate to HICs for perceived career advancement and potential financial advantages [12]. We envisage that students from both HICs and LMICs will work hand in hand in developing locally relevant project hypotheses, methods, and analyses. Senior academics and clinicians facilitating these partnerships could provide students with technical and mentorship support in planning and conducting research. Nurturing LMIC students’ research capabilities and confidence in such a manner may help combat the paucity of research activity that stems from the absence of research culture and training opportunities [13].

## NIHR global health research group for neurotrauma

One group that endeavours to integrate this model for international health electives within their health partnership is the National Institute for Health Research Global Health Research Group for Neurotrauma (NIHR GHRGN), based at the University of Cambridge.

This group is financed through UK Overseas Development Assistance funding and tasked with the mission to improve global neurotrauma care through a series of projects with a number of LMIC partners. The group aims to map, understand, and implement innovation in neurotrauma care, as well as measure and build research capacity within their partner institutions. At the heart of the group’s philosophy is engaging students interested in both global health and neurotrauma to dedicate their time into conducting high-quality academic research as part of existing projects. These enjoy the support of established academic partnerships and faculty supervision. Traditionally, students’ lack of experience in planning projects abroad means that international health electives are unstructured, resulting in a patchwork of poorly organized individual initiatives [14]. The group counters this by working with students in the design and management of projects, enabling ethical and safe research; projects are developed in concordance with senior academics from both Cambridge and LMIC institutions, and students have opportunities to work directly with local investigators and students at their LMIC institutions . To further the research capabilities and education of both the LMIC and HIC students, the group has partnered with the British Medical Journal to deliver online ‘Research to Publication’ courses. Coordinating multiple student international health electives over time and across varying locations, all with a shared purpose and training, allows the group to maximize synergies and lay out a research roadmap.

The early work of the NIHR GHRGN in engaging students has provided important insights for others seeking to emulate this example (see Appendix). Recruiting interested UK students has been straightforward, using formal teaching sessions as part of the undergraduate global health curriculum, local global heath conferences, and electronic communications through the University networks. HIC student projects are ongoing, involving all stages of the research development process including systematic review, hypothesis generation, and protocol design. International health electives are planned to support this work, facilitated through partnerships with relevant LMIC members of the GHRGN. Recruiting LMIC students has been more challenging in the early stages, while partner institutions establish their own research agendas and contractual and funding arrangements are finalized.

Whilst partnerships such as these are invaluable opportunities for students, training global healthcare professionals will also require structured Global Health teaching on surgery and anaesthesia [15]. This can be embedded into pre-clinical or clinical teaching, or taught as a standalone module. Educating students about the utility of global surgery and anaesthesia as public health tools can go far to correct the misconceptions regarding their cost-effectiveness and subsequent impact in LMICs. Indeed, many UK universities, including Cambridge, are now seeking to increase the global health content of their core curriculum. The challenge is then to embody the ideals proposed in this taught content within the practical experience of international health electives.

## Conclusion

The onus is on the international community to address the inequity and disparity in the burden of disease, both medical and surgical alike. The passion of today’s medical students can generate much needed momentum into this nascent field, driving home the importance of integrating global health into medical school curricula. The principles that underpin this teaching should suffuse international health electives, which some may argue are ethically questionable in their current form. We propose that the ethical oversight required by academic work and the partnership of equals needed to do research can help ensure electives are ethical, effective, and engaging. The NIHR GHRGN provides an example as to how these institutional partnerships can nurture the global surgeons and anaesthetists of tomorrow.

## Appendix

### Ten lessons from the NIHR GHRGN

1. The benefit of a dedicated awareness-raising program involving lectures, events, meetings, and electronic communications to make students aware of their opportunities within the Group
2. The need for a dedicated senior contact point for students within the Group to ensure they are paired with suitable projects and academics, mentor their progress, and advocate for their interests
3. The need for funding available within the Group to support the students’ research projects, such as publication fees for articles
4. The need for a funding strategy to support international health electives, such as internal and external bursary applications, or helping support writing these expenses into the initial funding application
5. The need for early planning of international health electives to accommodate competing research and undergraduate timetables
6. The benefit of integration with existing Global Health efforts; for example, the NIHR GHRGN is partnered with the Tropical Health and Education Trust and Cambridge Global Health Partnerships to support project development and help advise on travel, personal safety, and insurance issues
7. The need to encourage LMIC partners to identify students who would be interested in engaging with the group and to support them in co-developing research projects
8. The benefit of regular Group meetings to which all interested students are encouraged to attend and which LMIC partners attend via Skype
9. The difficulty for students in managing research projects alongside their undergraduate teaching, and the need for the Group to support them with this
10. The crucial role of a Group Program Manager in ensuring that student engagement remains at the heart of the Group’s activities
